# A positive genotype–phenotype correlation in a large cohort of patients with Pseudohypoparathyroidism Type Ia and Pseudo-pseudohypoparathyroidism and 33 newly identified mutations in the *GNAS* gene

**DOI:** 10.1002/mgg3.117

**Published:** 2014-12-04

**Authors:** Susanne Thiele, Ralf Werner, Joachim Grötzinger, Bettina Brix, Pia Staedt, Dagmar Struve, Benedikt Reiz, Jennane Farida, Olaf Hiort

**Affiliations:** 1 Division of Experimental Paediatric Endocrinology and Diabetes, Department of Paediatrics, University of LübeckLübeck, Germany; 2 Institute of Biochemistry, Christian-Albrechts-University of KielKiel, Germany; 3 Institute for Integrative and Experimental Genomics, University of LübeckLübeck, Germany; 4 Service de Pédiatrie 2 unité, Hôpital universitaire Abderrahim Harouchi Chu ibn RochdCasablanca, Morocco

**Keywords:** Albright hereditary osteodystrophy, G proteins, genotype–phenotype correlation, *GNAS*, Gs*α*, mutation, pseudohypoparathyroidism

## Abstract

Maternally inherited inactivating *GNAS* mutations are the most common cause of parathyroid hormone (PTH) resistance and Albright hereditary osteodystrophy (AHO) leading to pseudohypoparathyroidism type Ia (PHPIa) due to Gs*α* deficiency. Paternally inherited inactivating mutations lead to isolated AHO signs characterizing pseudo-pseudohypoparathyroidism (PPHP). Mutations are distributed throughout the Gs*α* coding exons of *GNAS* and there is a lack of genotype–phenotype correlation. In this study, we sequenced exon 1–13 of *GNAS* in a large cohort of PHPIa- and PPHP patients and identified 58 different mutations in 88 patients and 27 relatives. Thirty-three mutations including 15 missense mutations were newly discovered. Furthermore, we found three hot spots: a known hotspot (p.D190MfsX14), a second at codon 166 (p.R166C), and a third at the exon 5 acceptor splice site (c.435 + 1G>A), found in 15, 5, and 4 unrelated patients, respectively. Comparing the clinical features to the molecular genetic data, a significantly higher occurrence of subcutaneous calcifications in patients harboring truncating versus missense mutations was demonstrated. Thus, in the largest cohort of PHPIa patients described to date, we extend the spectrum of known *GNAS* mutations and hot spots and demonstrate for the first time a correlation between the genetic defects and the expression of a clinical AHO-feature.

## Introduction

Inactivating mutations in the Gs*α* encoding exons of *GNAS* result in Albright hereditary osteodystrophy (AHO) characterized by a variably expressed array of clinical features such as short stature, brachymetacarpia, subcutaneous ossifications, and mental retardation due to haploinsufficiency of Gs*α*. Tissue-specific imprinting leads to predominantly maternal expression of Gs*α* in the renal proximal tubules, thyroid, gonads, and pituitary gland. Thus, maternally inherited mutations cause, in addition to AHO features, resistance to parathyroid hormone (PTH) and other peptide hormones that mediate their action through G protein coupled signaling pathways, such as thyroid-stimulating hormone (TSH), growth hormone-releasing hormone (GHRH), and gonadotropin-releasing hormone (GnRH). AHO in combination with PTH resistance and a diminished in vitro measured activity of Gs*α* protein characterizes pseudohypoparathyroidism type Ia (PHPIa, MIM: 103,580), the most common subtype of the rare group of disorders summarized under the term pseudohypoparathyroidism (PHP). Paternally inherited mutations located at the paternal imprinted allele of *GNAS* lead to unaffected maternal *GNAS* expression in imprinted tissues. This condition is therefore characterized by isolated AHO features and has been termed as pseudo-pseudohypoparathyroidism (PPHP, MIM: 612463) (reviewed in Levine 2012; Linglart et al. 2013). Another *GNAS*-related disorder is the progressive osseous heteroplasia (POH, MIM: 166350), a rare autosomal dominant disorder inherited in most cases from the paternal allele. POH is characterized by dermal ossifications beginning in infancy, followed by increasing and extensive bone formation in muscle and fascia (Shore et al. 2002). Since AHO signs can be very variable (see also Table S1) and can include subcutaneous calcifications and since the same mutations can lead either to PHPIa or to POH it has been postulated that POH represents an extreme end of the spectrum of AHO features (Adegbite et al. 2008).

Heterotrimeric guanine-binding proteins (G proteins) are composed of an *α*, *β*, and *γ* subunit and are crucial mediators of signal transduction pathways of more than 1000 G protein coupled receptors (GPCRs) (Wettschureck and Offermanns 2005). Gs*α* is part of stimulatory G proteins acting via cyclic adenosine monophosphate (cAMP) and protein kinase A (PKA). When a ligand activates a GPCR, the receptor undergoes a conformational change leading to an interaction with the G protein. Thereby, a conformational change of the G protein is induced, resulting in a GDP/GTP exchange of Gs*α*. GDP release is the time-limiting step in this activation cycle. GTP binding leads to an extensive change of the tertiary structure mediated by three flexible switch regions, resulting in the dissociation of the *α*-GTP complex from the *βγ* subunits. Then, Gs*α*-GTP transmits signals to several downstream effectors, for example, leading to cAMP synthesis by adenylyl cyclase (AC). An intrinsic GTPase of the *α*-subunit, hydrolyzing bound GTP to GDP performs the turn-off mechanism and the subunits join together again (reviewed in Johnston and Siderovski 2007; Rasmussen et al. 2011). The Gs*α* protein contains a *α*-helical domain and a highly conserved guanine nucleotide-binding domain, also referred to as GTPase domain with high structural resemblance to the proto-oncogene ras. Both domains are connected by the switch regions I to III and together create the guanine-binding pocket (Sunahara et al. 1997). Interactions between both functional domains highly influence the GDP/GTP exchange and the following dissociation of the *α* and *βγ* subunit (Johnston and Siderovski 2007).

PHPIa and PPHP subtypes can be diagnosed by a diminished Gs*α* protein activity, investigating solubilized Gs*α* from patient-derived erythrocyte membranes, and by sequencing analysis of the *GNAS* gene. The Gs*α*-encoding *GNAS* gene (MIM 139,320) on chromosome 20q13.11 consists of 13 exons and 12 introns. Although the PHPIa phenotype can also occur in some cases due to epigenetic changes of the complex imprinted *GNAS* gene locus (de Nanclares et al. 2007; Mariot et al., 2008; Mantovani et al. 2010) and due to still unknown causes, in up to 70% of patients, inactivating *GNAS* mutations are found (Ahrens et al. 2001; Elli et al. 2013).

To date 193 inactivating *GNAS* mutations have been described, including missense, truncating, and splice site mutations, and small/large deletions or insertions. Most of them are summarized in the Human Gene Mutation Database (http://www.hgmd.cf.ac.uk/ac/all.php) or at the Leiden Open Variation Database (www.lovd.nl/GNAS). The mutations are distributed throughout the whole coding region of *GNAS* and only a recurring 4 bp deletion in exon 7 has been considered common among patients with AHO (Weinstein et al. 1992; Ahrens et al. 2001; Elli et al. 2013; Fernández-Rebollo et al. 2013). In 2001, we described *GNAS* mutations in a cohort of 21 PHPIa patients (Ahrens et al. 2001) and further cohorts of 13–53 patients with inactivating *GNAS* mutations have been reported (Aldred and Trembath 2000; Linglart et al. 2002; de Sanctis et al. 2003; Long et al. 2007; Elli et al. 2013; Fernández-Rebollo et al. 2013). However, based on the extremely variable position of mutations in the gene, it is difficult to predict a phenotype caused by a certain mutation in these cohorts.

In this study we analyzed the data from our molecular genetic analysis of 88 PHPIa and PPHP patients with identified inactivating *GNAS* mutations, and compared them to the clinical data. Furthermore, we discuss the putative functional consequences of the 15 newly identified missense mutations based on the functional domains of Gs*α*.

## Material and Methods

### Patients

We collected clinical information at the time of diagnosis and blood samples of patients with variable clinical features of AHO. From these samples, we performed sequencing analysis of *GNAS* as well as determination of Gs*α*-activity. In some patients (collected until June 2004), the result of the Gs*α* protein activity measurement has already been summarized in a cohort, but not in detail (Ahrens and Hiort 2006). All subjects or their guardians gave informed consent to the study. Studies were approved by the ethical committee of the University of Lübeck as part of the funded project on AHO (see acknowledgement). Features of AHO, biochemical results, in-vitro measured Gs*α* activity and corresponding *GNAS* mutations are summarized in Table S1.

### Biochemical investigations, Gs *α* protein activity, and *GNAS* mutation analysis

An endocrine evaluation included a survey of serum calcium, phosphate, and PTH levels. Determination of the laboratory diagnostics has been done in our laboratory or partly in the laboratories of the referring physicians using standard procedures. The activity of Gs*α* protein extracted from erythrocyte membranes of patients was investigated as described earlier (Levine et al. 1980; Ahrens et al. 2001). Results obtained in triplicate were expressed as percentage of the mean of healthy controls (normal range: 85–115%). For molecular genetic analysis, genomic DNA derived from peripheral leukocytes was isolated by standard procedures (Qiaquick DNA kit; Qiagen, Hilden, Germany). *GNAS* exons 1–13 (RefSeq NM_001077488.1) including all intron/exon boundaries were amplified in 11 fragments by PCR (primer sequences available upon request). PCR-amplified DNA was sequenced by direct cycle sequencing using the BigDye Terminator v1.1 Cycle Sequencing Kit (Applied Biosystems, Foster City, CA) and an ABI 3130 capillary sequencer (Applied Biosystems, Foster City, CA) (Thiele et al. 2011). Mutations were confirmed by sequencing of a second-independent PCR sample.

### Statistical analysis and structural analysis of Gs*α*

The relationship between molecular genetic changes and the clinical AHO signs has been investigated by using chi-square test. After Bonferroni correction, a *P*-value of less than 0.01 was deemed to indicate statistical significance. Analysis of the clinical variables included brachymetacarpia, short stature, obesity, mental retardation, and subcutaneous calcifications. Because of the known parental imprinting effect, we limited statistical analysis to patients with PHPIa, harboring the mutation on the maternal allele.

In our script numbering of the *GNAS* mutations is based on the reference sequence NM_001077488.1 also used in “The Leiden Open Variation Database.” However, since the “Human Gene Mutation Database,” most to date described *GNAS* mutations, and the description of the crystal structure of Sunahara et al. (1997) are based on the cDNA sequence RefSeq NM_000516.4 (differing in the sequence to the other reference by a differential spliced CAG localized between exon 3 and 4), most mutations are described differently in the literature (one amino acid less). +1 is the first nucleotide of the ATG translation initiation codon in the reference sequence.

Mutation description was checked with Mutalyzer software (https://mutalyzer.nl). For structural analysis the crystal structure of Gs*α* in complex with GTP*γ*-s (Sunahara et al. 1997, PBD number: 1AZT; http://www.rcsb.org) was used. The structural representations were generated using the RIBBONS software (Kraulis 1991). We annotated the newly identified variants using annovar (Wang et al. 2010). The data of the 1000 genomes project (1092 samples) (1000 Genomes Project Consortium 2012) and the exome sequencing project (6500 samples) (Exome Variant Server, NHLBI GO Exome Sequencing Project [ESP], Seattle, WA [URL: http://evs.gs.washington.edu/EVS/]) was used to check for the allele frequency of this variants. For the functional prediction we used the scaled Combined Annotation Dependent Depletion (CADD) score (Kircher et al. 2014), which describes the deleteriousness of single-nucleotide variants.

## Results

### *GNAS* mutation analysis

Our molecular genetic analysis revealed 58 distinct mutations in 88 unrelated patients and in 27 relatives (22 mothers and five sisters or brothers). Twenty-eight were missense, 14 were frameshift, and four nonsense mutations. In three samples one amino acid was deleted, in one sample one amino acid duplicated. Eight mutations were splice site mutations (Table S1). All newly identified mutations have been included into the Leiden Open Variation Database (http://www.lovd.nl/GNAS).

In 88 index patients, we identified 26 familial mutations, 24 of them proven inherited from the mother and 40 de novo mutations, meaning, the mother or the father, did not bear the mutation. In 22 index patients, we did not get material from family members for analysis, so the pattern of inheritance is unknown in these cases.

Thirty-three novel mutations, including 15 missense mutations, were found in 34 unrelated families (Table S1). We annotated the newly discovered variants using annovar (Wang et al. 2010) to check for the allele frequency. None of these variants have been detected in the 1000 genomes project (1092 samples) (1000 Genomes Project Consortium 2012) or the exome sequencing project (6500 samples) (Exome Variant Server, NHLBI GO Exome Sequencing Project [ESP], Seattle, WA, URL: http://evs.gs.washington.edu/EVS/) before.

Most of the concerned amino acids (L30, E169, L172, T205, M222, F239, E269, K294, and F346) are highly conserved in other G*α*-subclasses (G*α*i1, G*α*i3, G*α*t, G*α*12, G*α*13, G*α*q). R200 is conserved in all but G*α*12 and G*α*13, whereas V248 is conserved in all but not G*α*t. V118, A270, L283, and E328 are unique for Gs*α* (Johnston and Siderovski 2007). In addition, an alignment with Gs*α* orthologs of different species (rat, mouse, and human) showed that all affected residues are highly conserved. All nondisruptive mutations demonstrate a high CADD score (ranging from 14.1 to 36), meaning that the variants are predicted to be at least under the 5% most deleterious substitutions that you can do to the human genome (Kircher et al. 2014). Furthermore, all newly identified mutations were associated with a diminished Gs*α* protein activity.

The mutations were distributed throughout all Gs*α* coding exons. We detected 29 mutations in the *α*-helical domain of Gs*α*, five in the region between *α*-helical and ras-like domain, and 24 different mutations in the ras-like domain. The least affected region was the insertion one region encoded by exon 3, leading to four different splice variants of Gs*α*, in which only one inactivating mutation in a family has been described so far (Thiele et al. 2007). We confirmed the known deletion hotspot in exon 7, (p.D190MfsX14) (Weinstein et al. 1992; Ahrens et al. 2001; Elli et al. 2013) by identifying 15 affected patients and detected two novel hotspots, one in exon 6 (p.R166C), which concerned five of our patients and has been described several times before (Miric et al. 1993; Ahrens et al. 2001; Fernández-Rebollo et al. 2013) and another in the exon five acceptor splice site (c.435 + 1G>A), which concerned four of our patients and has also been described before (Wilson et al. 1994; Rickard and Wilson 2003; Aldred 2006).

### Variability of phenotypical and biochemical results

A total of 81 out of 88 index patients with heterozygous inactivating mutations were diagnosed with PHPIa (92%) and only six patients with PPHP (6.8%) based on the presence or absence of endocrinopathies. One patient (1.1%) with POH was identified. The age at diagnosis ranged from 1 week to 68.3 years and the mean of patients with truncating mutations was 8.75 years and in patients with missense mutations 13.06 years. Altogether, 42 of the index patients were male and 46 were female.

At the age of diagnosis, 59 of the 77 PHPIa patients of whom clinical data were available (76.6%) had brachymetacarpia, which was the most common clinical sign, followed by obesity (51 patients, 66.2%), mental retardation (40 patients, 51.9%), subcutaneous calcifications (29 patients, 37.7%, and short stature (25 patients, 32.5%).

In 74 of 81 PHPIa patients (91.3%) elevated PTH levels were found ranging from 56 to 913 pg/mL (reference range 15–55). 25 PHPIa patients had hypocalcemia (30.9%) with levels below 2.1 mmol/L (reference range 2.1–2.6). 41 PHPIa patients had hyperphosphatemia (50.6%) with levels higher than 1.75 mmol/l (reference range 0.9–1.75). The highest measured phosphate level was 3.39 mmol/L.

Comparing the phenotype of PHPIa patients harboring deleterious truncating mutations (only nonsense and frameshift mutations, which most probably cause a loss of protein activity, *n* = 33), with those patients harboring missense mutations (*n* = 31), we observed a highly significant difference on subcutaneous calcifications (*P* = 2.6e-06). In addition, a significant higher amount of brachymetacarpia in patients harboring missense mutations has been detected (*P* = 0.005).

Obesity (*P* = 0.34), short stature (*P* = 0.10), and mental retardation (*P* = 0.30) did not vary significantly (Table1).

**Table 1:** Results of statistical analysis of clinical AHO features in PHPIa patients harboring missense versus truncating mutations.

Clinical sign	Missense mutations (n = 33)	Truncating mutations (n = 31)	*P*-value
Brachymetacarpia	30 (90.9%)	19 (61.3%)	5.2e-03
Obesity	22 (66.7%)	24 (77.4%)	3.4e-01
Mental retardation	16 (48.5%)	19 (61.3%)	3.0e-01
Short stature	15 (45.4%)	8 (25.8%)	1.0e-01
Subcutaneous calcifications	2 (6.1%)	19 (61.2%)	2.6e-06

Result of statistical analysis of PHPIa patients comparing the occurrence of clinical signs in patients harboring missense mutations versus truncating mutations (*n*: numbers of patients). The occurrence of brachymetacarpia and subcutaneous calcifications is significantly different between both types of mutations.

Aside from subcutaneous calcifications and brachymetacarpia, the phenotype of our patients was highly variable revealing no visible genotype–phenotype correlation. This is especially seen in patients harboring the same mutation, such as patients with mutations in the hotspots. Even in patients with a similar age at diagnosis bearing a nonsense mutation or even the same nonsense mutation (Table S1), there is a broad range of phenotypical signs and an obvious lack of genotype–phenotype correlation. For example, comparing three patients with the p.D190MfsX14 mutation at a similar age at diagnosis (ranging from 14 years 5 month to 16 years and 8 month old), Patient P52 (14 years 5 months) had short stature, brachymetacarpia, and subcutaneous calcifications, while Patient P54 (16 years 8 months) had obesity, brachymetacarpia, mental retardation, and subcutaneous calcifications and the patient P53 (14 years 5 months, with POH) had only severe (sub)cutaneous and muscular calcifications (all results are summarized in Table S1) at the age of diagnosis.

## Discussion

The G protein/cAMP/PKA mediated signal transduction pathway is of high importance for growth, cell differentiation and metabolism due to extracellular ligands. The *α*-subunit of stimulatory G proteins Gs*α* is crucial for mediating these effects. In the present study, we report the positive results of the largest mutation screening of the *α* subunit of stimulatory G proteins described so far, leading to the identification of two new hotspots and 33 mutations that have not been reported before. Furthermore, we demonstrate for the first time a connection between the severity of the mutation and the phenotypical signs of subcutaneous calcifications and brachymetacarpia in patients with PHPIa.

In the following part we discuss the putative consequences of the newly identified missense mutations p.L30P, p.V118G, p.E169K, p.L172P, p.R200G, p.T205I, p.M222T, p.F239C, p.V248M, p.E269K, p.A270T, p.L283V, p.K294R, p.E328K, p.F346L, and the missense mutation p.R166C found in several patients based on the affected amino acid residues and the functional domains of Gs*α*.

The Gs*α* protein is composed of a *α*-helical domain containing *α*N, *β*1, and a six-helix bundle *α*A-*α*F (amino acid 53–199), and further of a highly conserved GTPase domain consisting of five *α* helices surrounding a six-stranded *β*-sheet (amino acid 225–395). The *α*-helical and GTPase domain are connected by two *β*-strands constituting the *β*2/*β*3-loop, called the interswitch, and by three flexible switch regions I to III, which create together the guanine-binding pocket (reviewed in Johnston and Siderovski 2007).

**Figure 1:**
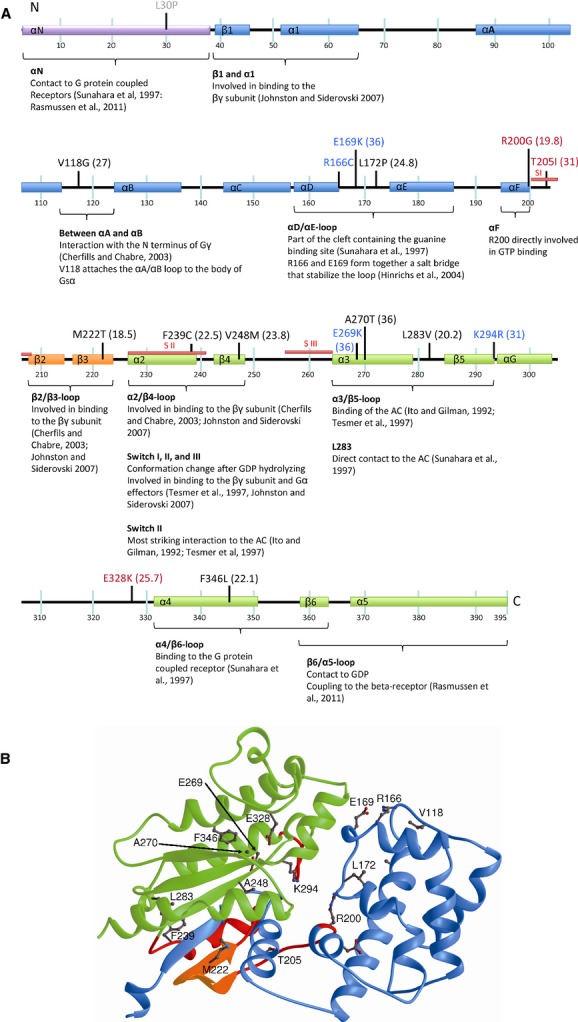
Localization of the 15 newly identified missense mutations and mutation R166C based on the structure of Gs*α*. (A) Scheme of the Gs*α* protein, its functional domains and relative locations of the newly identified missense mutations. Blue bars represent the *α*-helical domain, orange bars the GTP-binding domain, and green bars the ras-like domain. SI to III (in red) represents the three switch domains. Class I amino acids are written in black, class II in red and class III in blue. The CADD score is included in brackets. (B) Three-dimensional ribbon representation of Gs*α* demonstrates the structural interaction between the different domains. The *α*-helical domain is colored in blue, the interswitch region in orange, and the ras-like domain in green, both domains connected by the three switch regions shown in red. The amino acids concerned by newly identified missense mutations are accentuated. Since the first part of the protein has not been resolved in the X-ray structure, L30 is not depicted in the picture.

The *α*-helical domain contacts the GPCR (Rasmussen et al. 2011), is part of the cleft constituting the guanine-binding site (Sunahara et al. 1997), and is involved in binding to the *βγ* subunit (reviewed in Johnston and Siderovski 2007). The switch regions change their conformation upon GTP binding and interact together with *β*2, *β*3 with the *β*-subunit of G proteins (Cherfils and Chabre 2003). Furthermore, switch II directly interacts with the AC (Itoh and Gilman 1992; Tesmer et al. 1997). The ras-like-domain contains part of the binding site for the *βγ*-subunit (Cherfils and Chabre 2003; Van Eps et al. 2006), and part of the binding site for the AC (Itoh and Gilman 1992; Tesmer et al. 1997) and the GTPase (Sunahara et al. 1997). The carboxyl-terminal part of the ras-like domain interacts with the GPCR (Sunahara et al. 1997; Rasmussen et al. 2011). For the location of the newly identified missense mutations in combination with the functional domains of Gs*α* see Figure1A and B.

Since the 35 N-terminal amino acid residues are not resolved in the X-ray structure, the structural consequences of mutation p.L30P remains unclear. However, this region is in contact with the GPCR (Sunahara et al. 1997; Rasmussen et al. 2011), suggesting that mutation p.L30P may interfere with this interaction. All other mutations can be divided into three classes (Table2) not related to the phenotype of the patients, but rather to the affected amino acid residues and functional domains of Gsa.

**Table 2:** Classification of newly identified missense mutations.

Class	Affected amino acids
I	V118, L172, M222, F239, V248, A270, L283, F346
II	R200, T205, E328
III	R166, E169, E269, K294, E328

Affected amino acids of the newly identified missense mutations (and of the known mutation R166C) depending on the location of the amino acids. Class I amino acid residues are predicted to be involved in the hydrophobic core of the protein may lead to instability or even completely unfolding. Class II amino acid residues are involved in cofactor binding, while class III amino acid residues are involved in formation of salt bridges and mutations are also predicted to lead to instability of the protein structure.

The first class affects amino acid residues involved in the hydrophobic core of Gs*α*, in which mutations are predicted to lead to instability or even completely unfolding of the protein. V118 is part of the hydrophobic core of the protein and attaches the *α*A/*α*B loop to the body of the molecule. Replacing the valine by a glycine (p.V118G) may lead to a detachment of the *α*A/*α*B loop resulting in an increased flexibility and a decreased stability of the protein. L172 lies between *α*D and *α*E and the mutation p.L172P may influence the relative orientation of the adjacent helices. An introduction of the much smaller side chain of a threonine in p.M222T leads probably to a reorientation of the adjacent *β*-strands resulting in a loss of GTP binding. Reducing the size of the side chain in the mutant p.F239C (in *α*2) or introduction of the much bigger side chain of methionine in p.V248M (in *β*4) will also lead to strong alterations of the overall structure. Since the region between *α*2 and *α*3 (including *β*4) serves as a universal site for effector engagement (reviewed in Johnston and Siderovski 2007), the mutations may directly impair the AC binding. For L283 Sunahara et al. described a direct contact to the AC (Sunahara et al. 1997). Thus, reducing the size of the hydrophobic side-chain in the mutant p.L283V may lead to direct changes of the ability to activate the AC. F346 is part of a cluster of aromatic amino acid side chains (F274 and F247) and a substitution into a leucine (p.F346L) will destroy this cluster important for the overall stability of the protein. A270 is also located in the hydrophobic core of the protein and replacement by the bigger and hydrophilic side chain of a threonine (p.A270T) will lead to destabilization of the protein (Fig.1A and B).

The second class affects amino acid residues involved in cofactor binding. Mutations belonging to this class will abolish cofactor binding resulting in an inactive protein.

Inspection of the three-dimensional structure of the Gs*α* protein reveals that R200 in helix *α*F is directly involved in binding the GTP and a mutation into a glycine residue (p.R200G) leads to an abrogated GTP binding. Since T205 is part of a hydrogen bond network responsible for Mg^2+^ binding, the mutation p.T205I will also lead to a distortion of the GTP binding. E328 binds directly a phosphate group. Reversal of the charge as in p.E328K will inhibit this interaction and destabilize the structure as well as the GTP binding.

Class III mutants affect amino acid residues involved in forming salt bridges (R166, E169, E269, K294, E328) and are predicted, like class I, to lead to instability of the overall protein structure. Mutation p.R166C has been described several times before (Miric et al. 1993; Ahrens et al. 2001; Fernández-Rebollo et al. 2013), while p.E169K was newly identified in this study. R166 forms a salt bridge with E169 that stabilizes the *α*D/*α*E-loop localized at the top of the cleft formed between the *α*-helical domain and the GTPase domain in which the guanine-binding site is deeply buried. By functional analysis of R166 and E169, Hinrichs showed that the mutants p.R166A and p.E169A exhibited an increase in GTP binding and a higher GDP dissociation rate both resulting in an augmented capacity to activate the AC (Hinrichs et al. 2004). Interestingly, exchange into cysteine or lysine, respectively, leads to the opposite effects. Therefore, the mutations p.R166C and p.E169K seem to stabilize the *α*D/*α*E-loop in its closed form, thereby decreasing the GTP binding and lowering the GDP dissociation rate. E269 has been identified to form a salt bridge with R232, which is important for the interdomain interactions between switch II and III, as well as receptor catalyzed activation, which is destroyed in our mutant p.E269K. A salt bridge between K294 in the *β*5 region and D174 form a further important connection between the *α* helical domain and the GTPase domain, which is required for proper positioning of the two domains playing an important role in receptor activation (Codina and Birnbaumer 1994). Since in our mutant p.K294R this salt bridge is destroyed, sufficient receptor activation is disturbed. E328 is located in the *α*G/*α*4-loop and is forming a salt bridge with R375. Reversal of the charge as in p.E328K will inhibit this interaction and destabilize the structure as well as the GTP binding.

In this study we demonstrate for the first time a connection between the severity of the molecular genetic defect and the presentation of subcutaneous calcifications in patients with PHPIa. Subcutaneous calcifications are defined as de novo formation of islands of heterotopic bone in skin and subcutaneous fat (Shore et al. 2002). Recently, Pignolo et al. (2011) demonstrated that Gs*α* is a key regulator of adipose-derived mesenchyme cells in mice and that inactivation of *Gnas* exon 1 results in deregulation and decreased expression of multiple *Gnas* transcripts correlated with accelerated osteoblast differentiation of adipose cells. Our data are in agreement with three further studies: First, Adegbite and colleagues found in 42% of their patients with subcutaneous calcifications (including PHPIa, PHPIc, and POH) the truncating mutation p.D190MfsX14 in *GNAS* (Adegbite et al. 2008). Second, Lebrun reviewed 20 different inactivating *GNAS* mutations in POH patients, from whom 16 (80%) were truncating and only 1 was a missense mutation (Lebrun et al. 2010). And third, in a recently published study Fernández-Rebollo demonstrated subcutaneous calcifications only in AHO patients harboring mutations in Gs*α* coding exons of *GNAS*, but not in AHO patients harboring *GNAS* epigenetic defects (Fernández-Rebollo et al. 2013). These data suggest, that the complete loss of Gs*α* from one allele, enhance the probability of subcutaneous calcifications, may be due to the additional loss of alternative *GNAS* transcripts (Pignolo et al. 2011).

Since the occurrence of brachymetacarpia in PHP patients is age dependent, we interpret the higher occurrence of brachymetacarpia in patients harboring missense mutations due to the later age at diagnosis in this patient group, compared to patients harboring truncating mutations.

Except for subcutaneous calcifications and brachymetacarpia, we confirm the variation of AHO features and their occurrence with a lack of genotype–phenotype correlation (Elli et al. 2013; Fernández-Rebollo et al. 2013) in patients harboring the same mutation such as p.D190MfsX14 or p.R166C. Because some AHO features are not developed before a certain age, this observation may partly be explained by the different age at diagnosis. However, based on the observation that even patients with nonsense mutations with a similar age at diagnosis demonstrate a variable clinical phenotype, our results indicate that modifying factors other than residual Gs*α* function (such as epigenetic modifications due to environmental factors or different regulation of cofactors of the cAMP coupled pathway) may play a role in the strength of the clinical phenotype. To find a predictive phenotype for distinct mutations the more detailed analysis of a very large cohort of patients, of epigenetic modifiers, and the elucidation of possible cofactors may be helpful.

To summarize, in our study we present the phenotypical and genetic characterization of the largest cohort of PHPIa and PPHP patients described to date and enlarges the knowledge about pathogenic inactivating mutations in the high complex *GNAS* gene locus. Moreover, our study reveals for the first time a correlation between the severity of the mutation and the occurrence of subcutaneous calcifications and of brachymetacarpia, latter in which time modifiers may play a basic role.

## Supporting Information

**Table S1.** Genotype and phenotype of patients with PHPIa, PPHP, and POH harboring inactivating mutations in the *GNAS* gene. Table S1 summarizes age at diagnosis, sex, PHP subtype, AHO signs, laboratory diagnostics, Gs*α* protein activity, endocrinopathies, the identified *GNAS* mutation on nucleotide and on peptide level (based on the RefSeq NM_001077488.1) and the pattern of inheritance of our patients with PHPIa or PPHP, respectively.
